# Case report: A collision tumor of clear cell renal cell carcinoma and clear cell papillary renal cell tumor

**DOI:** 10.3389/fonc.2024.1284194

**Published:** 2024-02-28

**Authors:** Yingsheng Lin, Jinan Guo, Zaishang Li, Zengqin Liu, Jing Xie, Junxu Liu, Hongtao Jin, Kefeng Xiao

**Affiliations:** ^1^ The Second Clinical Medical College of Jinan University, Shenzhen, China; ^2^ Department of Urology, Shenzhen People’s Hospital (The Second Clinical Medical College, Jinan University, The First Affiliated Hospital, Southern University of Science and Technology), Shenzhen, China; ^3^ Department of Pathology, Shenzhen People’s Hospital (The Second Clinical Medical College, Jinan University, The First Affiliated Hospital, Southern University of Science and Technology), Shenzhen, China

**Keywords:** collision tumor, clear cell renal cell carcinoma, clear cell papillary renal cell tumor, case report, specimens

## Abstract

We report the case of a 51-year-old woman who was initially hospitalized in the respiratory department with cough and fever. Urinary computed tomography (CT) showed two different incidental masses in the right kidney. The patient underwent a radical right nephrectomy without lymph node dissection and postoperative adjuvant treatment. The pathological examination of the surgical specimens showed a collision tumor composed of a clear cell renal cell carcinoma (CCRCC) and a clear cell papillary renal cell tumor (CCPRCT). To the best of our knowledge, this is the first such case reported to date. No recurrence of local or distant metastasis was found during routine follow-up 14 months after the operation.

## 1 Introduction

Renal carcinoma is a common tumor of urinary system. In 2016, the World Health Organization (WHO) added a relatively rare subtype of renal cell carcinoma, clear cell papillary renal cell carcinoma (1), and in 2022 renamed it clear cell papillary renal cell tumor (CCPRCT) to more accurately reflect its inactive biology (2). A collision tumor is a special tumor type in which two or more tumors of different origins occur in the same tissue or organ. Primary renal collision tumors are rare, and there have been few reports of them. Here, we report a case of renal collision tumor composed of a clear cell renal cell carcinoma (CCRCC) and a CCPRCT. To the best of our knowledge, this is the first report of a renal collision tumor composed of a CCRCC and a CCPRCT.

## 2 Case description

**Figure 1:**
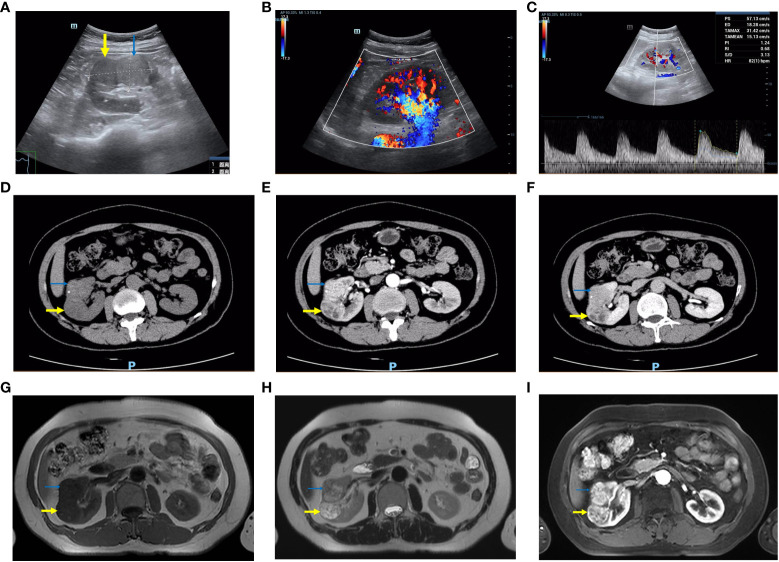
Imaging features of the right renal lesion. The thick yellow arrow is the CCRCC and the thin blue arrow is the CCPRCT. **(A–C)** Color ultrasound. **(A)** The boundary between the two masses is clear. **(B)** The blood flow signals were abundant. **(C)** High-speed arterial spectrum. **(D–F)** CT. The density of the CCRCC was lower than that of the CCPRCT in plain scan, and the CCPRCT had some calcifications. Both masses were obviously enhanced in the arterial phase. The CCRCC has cystic areas. The enhancement degree of the two masses decreased during excretion. **(G–I)** MRI. **(G)** T1WI **(H)** T2WI **(I)** Contrast-enhanced scan.

A 51-year-old Chinese woman was hospitalized in the respiratory department of our hospital because of fever and cough. Her previous history included anemia, craniocerebral surgery and blood transfusions during childbirth. She had neither a cancer history nor a family history of cancer. In the subsequent whole-body examination, urinary ultrasound found that there were two masses with a clear boundary (**Figures 1A–C**) in the middle parenchyma of the right kidney with sizes of approximately 38 × 28 mm and 36 × 29 mm respectively. The patient had no urinary system symptoms, lower back pain, or fever, and had not been exposed to hazardous chemicals or radioactive substances.

## 3 Diagnostic assessment

Computed tomography (CT) examination of the urinary system showed two round-like uneven density masses (**Figures 1D–F**) in the middle of the right kidney, with clear boundaries of approximately 32 × 28 mm and 36 × 25 mm, respectively. The patient was transferred to our department for further examination and treatment. Magnetic resonance imaging (MRI) revealed that most of the masses showed long T1 and T2 signal intensity (**Figures 1G–I**), small cystic changes could be seen locally, diffusion was not limited, and no clear signal decreased area was found in the inverse phase and fat compression sequence. Laboratory examination showed a hemoglobin concentration of 99.00 g/L, platelet count of 380000 cells/μL, and serum triglyceride of 2.79 mmol/L. The routine urine and renal function test results were normal.

**Figure 2:**
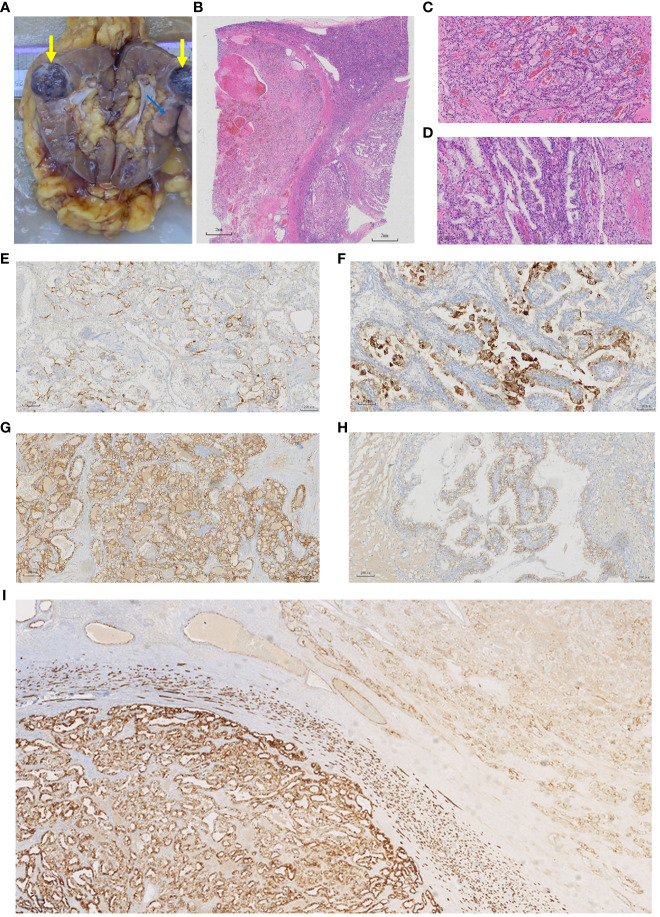
Pathological features of the specimen. **(A)** General appearance of the specimen. The taupe area (thick yellow arrow) corresponds to the CCRCC and the grey area (thin blue arrow) corresponds to the CCPRCT. **(B–D)** Histologic features of the specimen. **(B)** In the lower left part of the image is the CCRCC, the lower right part is the CCPRCT. **(C)** The CCRCC showed acinar and vesicular arrangement. (H&E, x10). **(D)** The CCPRCT showed branched tubular and papillary arrangement. (H&E, x10). **(E-I)** Immunohistochemical results. **(E)** CD10 (x10) membrane-positive in the CCRCC region. **(F)** CD10 (x10) cytoplasm-positive in the CCPRCT region. **(G)** CA9 (x10) intact membrane-positive in the CCRCC region. **(H)** CA9 (x10) Cup-shaped positive in the CCPRCT region. **(I)** KRT7 (x4) The CCRCC region (left side of the picture) was partially positive, while the CCPRCT region (right side of the picture) was diffusely positive.

The patient underwent robot-assisted laparoscopic radical nephrectomy without lymph node dissection. Two well-defined tumors were seen in the resected surgical specimen (**Figure 2A**) on the upper part of the kidney. One was grayish brown, 3.2 cm × 2.5 cm × 2 cm in size, while the other was a gray-white solid mass, 2.5 cm × 2 cm × 2 cm in size, with a moderate texture. By light microscopy, two tumors with different histological structures were observed (**Figures 2B–D**), of WHO/ISUP grade 2. The larger tumor showed acinar and nest-like structures; the centers of the acinar structures were filled with eosinophilic serous fluid or red blood cell casts; the tumor cells had eosinophilic cytoplasm and the nucleolus was not prominent. The surrounding stroma had a rich small vessel network. The smaller tumor contained branched tubular and papillary structures, and the tumor cells demonstrated eosinophilic cytoplasm. The nuclei did not have prominent nucleoli, and they tended to be located toward the luminal surface. The stroma was fibrous or smooth muscle-like. Both tumors were confined to the renal parenchyma, and there were no signs of tumor invasion in the perirenal adipose tissue, renal sinus adipose tissue, renal veins and branches, or renal pelvis. No cancer cells were found at the incisal margin of the ureter or the renal vein. Therefore, according to the 8th edition of the American Joint Committee on Cancer staging system, the tumors were staged as T1bNxMx.

Immunohistochemical analysis showed that the larger tumor was positive for FH and SDHB; partially positive for KRT7, vimentin and PAX8; positive for CD10 on cell membranes; positive for CA9 on intact cell membranes; with some cells positive for KIT; and negative for ALK, CK2, P504S, and GATA3. The small tumor was positive for FH and SDHB; diffusely positive for KRT7; partially positive for vimentin and PAX8; showed cytoplasmic CD10; positive for cup-shaped CA9; positive for KIT; and negative for ALK, CK20, P504S, and GATA3 (**Table 1**; **Figures 2E–I**). Fluorescence *in situ* hybridization (FISH) showed that 24% of the larger tumor had a 3p chromosome deletion, whereas the smaller tumor did not. The FISH data supported the morphological and immunohistochemical findings that two different tumor components were coexisting in the same lesion. Based on these findings, we believe that this was a renal collision tumor composed of a CCRCC and a CCPRCT. The patient recovered well postoperatively and did not undergo any radiotherapy or chemotherapy. To date, the patient has been followed-up for 14 months, and there have been no signs of local recurrence or distant metastasis.

**Table 1:** The difference in immunohistochemical results between the two tumors.

	CA9	CD10	KRT7
CCRCC	Intact membrane+	Membrane +	Part+
CCPRCT	Cup-shaped+	Cytoplasm +	Diffuse+

## 4 Discussion

Here, we describe the clinical, histological, and molecular features of an extremely rare collision tumor. As confirmed by immunohistochemistry and molecular analyses, this patient was diagnosed with having a renal collision tumor comprising a CCRCC and a CCPRCT.

Collision tumors are tumors of different tissue origins that coexist in the same tissue or organ without histological fusion (3). Collision tumors have been reported in different parts of the body, including the skin, thyroid, uterus, brain, gastrointestinal tract, and genitourinary tract (4–6). Primary renal collision tumors are rare, and only sporadic cases have been reported in the medical literature. Collision tumors can be characterized as a combination of benign-benign, benign-malignant, or malignant-malignant tumors. More than ten patients of renal collision tumors composed of two types of malignant tumors have been reported; however, renal collision tumors composed of a CCRCC and a CCPRCT have not been reported.

Collision, compound, and synchronous tumors must be differentiated. Complex tumors are also composed of two tumors with different morphology and immunohistochemistry, but they have obvious cell mixing and common driving mutations, showing histological differences of common origin, and having no clear boundaries between the tumor types (7). In a synchronous tumor, two different tumors occur at the same time, or in succession no more than two months apart; they are independent of each other and have a clear boundary (8). Therefore, collision tumors can be composed of synchronous tumors and vice versa. In this case, the two renal tumors were clear cell renal cell carcinoma and clear cell papillary renal cell carcinoma, without histological fusion and were well defined; therefore, they were identified as collision tumors.

Renal collision tumors, comprised of two types of malignant tumors, are extremely rare. The previous 16 patients of renal collision tumors all consisted of two malignant tumors of renal origin. This is the 17th reported patient and the first patient of a renal collision tumor comprising a CCRCC and a CCPRCT. There were some interesting observations from the previous cases (**Table 2**). First, except for one patient who was 24 years old, all previously reported patients were over 45 years old, which is similar to the age distribution of malignant renal tumors. Second, the male to female ratio of renal cell carcinoma is approximately 2:1, and the male-to-female ratio of these 17 patients of renal collision tumors is close to this value (11:6). Of course, because the number of tumors was too small, accidental occurrence could not be ruled out. Third, most patients are inadvertently diagnosed with no obvious clinical symptoms; some have abdominal pain, hematuria, and other symptoms. Finally, the current evidence suggests that the clinical prognosis of collision tumors may be affected by the subtypes and pathological stages of the more aggressive tumors.

**Table 2:** Clinical characteristics of cases with collision renal tumors combining two malignant components.

References	Age (y)/Sex	Symptoms	Tumor 1	Tumor 2	Treatment	Follow-up period
1− Anani et al. (7)	81/M	NA	CRCC	PRCC	RN	NA
2- Zhang et al. (9)	63/F	Hematuria	PRCC	CRCC	RN	NED at 20 months
3- Burch-Smith et al. (10)	67/F	Abdominal pain	CCRCC	CDC	RN+CT	Metastases at 5 months
4- Elian & Lam (11)	66/M	Hematuria	PRCC	RMC	RN+TT	NA
5- Kawano et al. (12)	64/F	Abdominal pain, fever	CRCC	CDC	RN+RT+CT	Metastases at 8 months
6- Bartos et al. (13)	70/M	NA	CCRCC	PRCC	RN	NED at 36 months
7- Matei et al. (14)	70/M	Abdominal pain, hematuria	CDC	PRCC	RN	NED at 57 months
8- Cho et al. (15)	24/M	Accidental finding	CCRCC	CDC	RN	NA
9- Gong et al. (16)	72/M	Weight loss, asthenia, anemia	CRCC	CDC	RN	NA
10- Roehrl et al. (17)	65/F	Accidental finding	CRCC	PRCC	RN	NED at 4 months
11- Moe et al. (18)	51/M	Accidental finding	PRCC	CCRCC	PN	NA
12- Salazar-Mejia et al. (19)	47/M	Dry cough, weight loss	CCRCC	CDC	RN+TT	NED at 12 months
13- Lamprou et al. (20)	68/M	Accidental finding	CCRCC	PRCC	RN	NA
14- Compérat (21)	67/F	Accidental finding	CCRCC	PRCC	RN	NA
15- Belle et al. (22)	64/M	Accidental finding	CCRCC	PRCC	RN	NED at 20 months
16- Belle et al. (22)	59/F	Accidental finding	CCRCC	CDC	EN	NED at 12 months
17- Current case	51/M	Fever, cough	CCRCC	CCPRCC	RN	NED at 14 months

CRCC, chromophobe renal cell carcinoma; PRCC, papillary renal cell carcinoma; CDC, collecting duct carcinoma; CCRCC, clear cell renal cell carcinoma; RMC, renal medullary carcinoma; CCPRCC, clear cell papillary renal cell carcinoma; NA, not available; RN, radical nephrectomy; PN, partial nephrectomy; EN, enlarged nephrectomy; CT, chemotherapy; RT, radiation therapy; TT, targeted therapy; L, left; R, right; NED, no evidence of disease.

To our knowledge, this is the first case report of a primary collision tumor comprising a CCRCC and a CCPRCT. There are challenges in determining the prognosis and clinical management of this type of patient. On the one hand, CCRCC is the most common subtype of renal cell carcinoma (23), which is prone to bleeding, cystic degeneration, necrosis, and calcification. In addition, CCRCC can easily invade the surrounding structures, often has a tumor thrombus in the renal vein and inferior vena cava, and is prone to lymph node metastasis, resulting in a poor prognosis. At the same time, renal collision tumors, involving two malignant tumors, most commonly include clear cell carcinoma [59% (10/17) of the cases shown in **Table 2**].

In contrast, CCPRCT is the fourth most common subtype of renal cell carcinoma after CCRCC, PRCC, and chromophobe cell renal cell carcinoma (2). In general, CCPRCT is small, with an average size of 2.6 cm (24). CCPRCT can be divided into solid and cystic types, and the solid type can evolve from the combined cystic type. The solid-enhanced CT scan was mainly in the fast-in and fast-out modes, and a few lesions showed progressive enhancement. Contrast-enhanced CT scans of the cystic type mainly show septal enhancement and nodular enhancement of the cyst wall (25–27). They are typically wrapped in a clear, thin, fibrous capsule. The sections are changeable, often pink, often accompanied by cystic changes, and there are generally no secondary changes such as bleeding, necrosis, or calcification. By microscopy, CCPRCT is usually well defined and has a fibrous capsule. The overall structural pattern can be tubular, papillary, solid, cystic, acinar, or a combination of these (28). At present, the prominent branched tubular structure and small papillary structure protruding from the capsule are considered the characteristic tissue morphologies of CCPRCT. Another feature is that the nucleus is usually located toward the luminal surface and away from the basement membrane, that is, it has reverse polarity (29). It is worth noting that CCPRCT has an inert clinical behavior, and most cases with TNM staging are pT1NOM0; thus far, there have been no reports of recurrence or metastasis (30).

When the morphological features are unclear, immunohistochemical staining is very important for the accurate diagnosis of CCPRCT. Diffuse expression of cytokeratin 7 is a characteristic marker of CCPRCT and the most important diagnostic criterion. In addition, the positive cup-shape staining of carbonic anhydrase IX and the negative expression of the renal cell carcinoma markers, CD10 and AMACR, are also important immune markers (28). It is worth noting that E-cadherin, vimentin, and β-catenin are often positive in CCPRCT, while simultaneous positivity of these three proteins is rarely seen in other renal cell carcinomas (2, 31).

Unlike other types of renal cell carcinoma, current studies have not found consistent genetic changes in CCPRCT; however, most cases show that the tumor does not have a 3p chromosome deletion, VHL gene mutation, or VHL promoter methylation (32). At the same time, there was no acquisition of chromosomes 7 and 17 or deletion of the Y chromosome in papillary renal cell carcinoma (PRCC) (31).

CCPRCT is a clinically inert tumor; therefore, correct diagnosis is very important for the treatment plan and the impact on the life of patients. Owing to morphological overlap, CCPRCT is easily misdiagnosed as other subtypes of renal cell carcinoma, such as CCRCC, PRCC, and Xp11 translocation RCC. This was also the key to the diagnosis of renal collision between CCRCC and CCPRCT in this case. Microscopically, CCRCC has a characteristic fine sinus vascular network, lacks the branched tubular structure of CCPRCT, and complex clear cell streamers, including bleeding, necrosis, cystic degeneration, and calcification in tumors, and the nucleus can be of a high grade (Fuhrman3-4 grade). Immunohistochemistry of CCRCC usually shows diffuse expression of CD10, CAIX, AE1/AE3, CAM5.2, EMA, PAX8, PAX2, and vimentin and a lack of expression of KRT7, AMACR, and 34bE12 (33, 34). Most CCRCC exhibit VHL mutations, promoter methylation, or deletion of the short arm of chromosome 3 (2, 25).

Microscopically, the typical features of PRCC are nipples, foamy histiocytes, and gravel along the axes of fibrous vessels. Immunohistochemical staining revealed that KRT7, CD10, AE1/AE3, CAM5.2, EMA, vimentin, and renal cell carcinoma markers AMACR, and 34bE12 were positive, whereas CA9 was negative (35). PRCC is usually associated with the acquisition of chromosome 7 or 17 and the loss of the Y chromosome (2).

Xpl1.2 translocation/TFE3 gene fusion-related renal cell carcinoma is a rare type of renal cell carcinoma that mainly occurs in children and adolescents. The most common structural pattern of this tumor is papillary or nesting with transparent epithelioid cells and rich sand calcifications. The nucleus was usually advanced, and the cytoplasm was granular or eosinophilic. Tumors usually show positive staining for cathepsin K, PAX8, CD10, E-cadherin, AMACR, and RCC antigens, but weak or no expression of AE1/AE3, CAM5.2, KRT7, and EMA (2). The most diagnostically significant feature is the translocation of the Xpl1.2 chromosome and the fusion of the TFE3 gene.

The origin of collision tumors and the relationship between the different types of tumors are not clear. Researchers have put forward various theories, but there is no clear consensus. Thus far, a variety of mechanisms have been proposed for the pathogenesis of collision tumors. First, it is completely accidental that two different tumors occur in the same anatomical site and are caused by different carcinogenic stimuli (36). Second, common carcinogenic stimuli lead to two different tumor phenotypes, that is, two different cell lines proliferate at the same time after co-carcinogenic stimulation from the microenvironment, resulting in two different phenotypes of tumors (10). Third, two different tumors differentiate from the same precursor cells. This theory suggests that the occurrence of colliding tumors may be related to the ability of cancer stem cells to differentiate into different tumor cell lines in the same organ or anatomical site (12). Finally, the occurrence of the first kind of tumor changes the microenvironment of organs or tissues, thus promoting the formation and spread of other tumors. This will increase the possibility of another primary tumor, resulting in a different phenotype of the second tumor (36). Of course, these theories may be a reasonable explanation for the collision tumor phenomenon.

Due to the rarity of renal collision tumor, its clinical impact has not been determined, and its treatment is currently based on standard guidelines for classic renal tumors. In our case, CCRCC and CCPRCT collided at the same site, and no local or distant metastasis was discovered by preoperative imaging examination, so surgery is a priority. Regarding the choice of surgical methods, considering that there are two tumors, considering that there are two tumors, and we do not know their specific properties, to completely remove the tumor, we chose radical nephrectomy, and completed the operation with the assistance of a surgical robot.

The prognosis of renal tumors with different histological subtypes is also different. As can be seen from **Table 2**, the survival time of collision tumors composed of renal collecting duct carcinoma is very short, while that of renal clear cell carcinoma is longer. In addition, the prognosis is likely to be related to the most invasive components with the highest nucleolar grade and stage. In our case, it was a collision tumor composed of CCRCC and CCPRCT, with WHO/ISUP grade 2 and AJCC stage T1bNxMx. Therefore, this patient may be able to obtain a better clinical prognosis.

In this case, robot-assisted laparoscopic radical nephrectomy was performed. We comprehensively analyzed the imaging and pathology results and did not schedule the patient for preoperative or postoperative radiotherapy or chemotherapy. The follow-up time and frequency of collision tumors should also be determined according to the subtype, grade, and stage of the tumor. Additionally, the patients were reexamined at the 5th and 10th month after operation and returned to the hospital for color ultrasound or CT at the 14th month (**Supplementary Figures 1**-**3**). Follow-up for 14 months showed no tumor recurrence or progression.

## 5 Conclusion

In summary, we report a collision tumor consisting of a CCRCC and a CCPRCT. To the best of our knowledge, this is the first such case reported to date. When two different histological components of CCRCC and CCPRCT are identified, it is important to consider the possibility of a collision tumor and to make a diagnosis by carefully examining the histological features and using immunohistochemical methods.

Based on our case and literature review, a renal collision tumor composed of two kinds of malignant tumors is very rare, and the first case is renal collision tumor composed of CCRCC and CCPRCT. The pathogenesis of this rare entity is largely unknown. Due to the lack of specific symptoms and radiological features, preoperative diagnosis is difficult, and more cases need to be recorded to better determine the treatment. In addition, more combined histological studies can obtain more information about its histogenesis, prognosis and the most appropriate treatment. Finally, the case still needs to be followed up for a longer time to determine the effect and prognosis of the treatment.

**Preprint**

A previous version of this manuscript was published as a preprint (37).

## Data availability statement

The original contributions presented in the study are included in the article/**Supplementary Material**. Further inquiries can be directed to the corresponding authors.

## Ethics statement

Written informed consent was obtained from the individual(s) for the publication of any potentially identifiable images or data included in this article.

## Author contributions

YL: Writing – original draft. JG: Formal analysis, Writing – review & editing. ZSL: Data curation, Writing – review & editing. ZQL: Data curation, Writing – review & editing. JX: Software, Writing – review & editing. JL: Investigation, Writing – review & editing. HJ: Writing – review & editing. KX: Writing – review & editing.
