# Supplementary material for: Case report: A collision tumor of clear cell renal cell carcinoma and clear cell papillary renal cell tumor
Source: Front Oncol. 2024 Feb 28;14:1284194. doi: 10.3389/fonc.2024.1284194 (PMC10933076; doi:10.3389/fonc.2024.1284194)
Supplement: Supplementary file 1 [file DataSheet_1.pdf]

Supplementary Material

1.1 Supplementary Figures

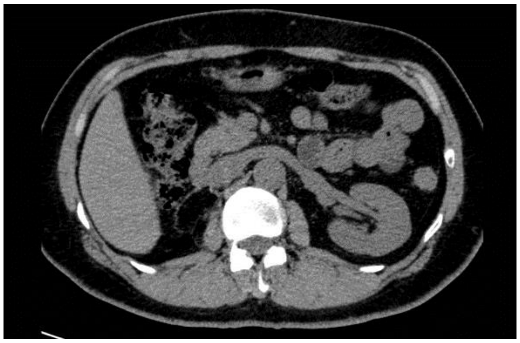

Supplementary Figure 1. Follow-up CT at 5 months after operation.

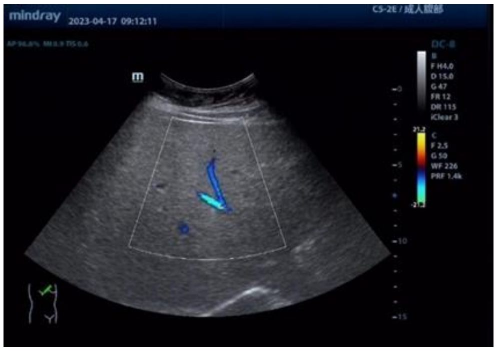

Supplementary Figure 2. Ultrasound, 10 months after operation.

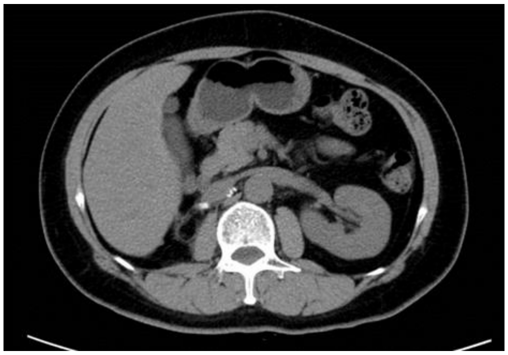

Supplementary Figure 3. Follow-up CT at 14 months after operation.
